# Children's behavioural problems and its associations with socioeconomic position and early parenting environment: findings from the UK Millennium Cohort Study

**DOI:** 10.1017/S2045796020000700

**Published:** 2020-08-13

**Authors:** K. Tamura, J. Morrison, H. Pikhart

**Affiliations:** Research Department of Epidemiology and Public Health, University College London, 1-19 Torrington Place, London, WC1E 6BT, UK

**Keywords:** Adolescence, child psychiatry, economic issues, families, social environment

## Abstract

**Aims:**

To investigate behavioural problems throughout childhood and adolescent, and its relationship with socioeconomic position (SEP) and early parenting environment.

**Methods:**

Using data from the Millennium Cohort Study conducted in the UK, behavioural problems of 14 452 children were analysed using a growth curve model. The children were followed from birth to adolescence, and their behavioural problems were measured by the Strengths and Difficulties Questionnaire (SDQ). The SDQ was sub-scaled into externalising and internalising problems. After assessing the general trajectory of children's behavioural problems, variables representing SEP and parenting environments were introduced to the model to analyse the association with children's outcomes.

**Results:**

Overall, children's trajectories in externalising problems showed a decreasing trend while internalising problems increased as they aged. Household income and maternal education in early childhood were independently associated with children's behavioural problems, while the association for maternal occupation was significantly weaker. Positive early parenting environments attenuated the association between SEP and children's behavioural problems. Also, with regards to children's behavioural problems, positive parenting explained more variance between children compared to SEP. Favourable parent–child relationship buffered the income gradient in children's behavioural problems during early childhood, and although this buffering effect did not last until adolescence, those who had good parent–child relationships developed better outcomes regardless of their SEP.

**Conclusions:**

The results of the study emphasise the importance of a positive early parenting environment for improving and reducing the socioeconomic gap in children's behavioural problems and encourages policies to promote better parenting circumstances.

## Introduction

In 2017, one in eight (12.8%) 5–19 year olds in the UK met the criteria for a mental disorder, and the prevalence of these disorders has kept an upward trend since the late 1990s (Sadler *et al*., 2018). It has also been shown that half of all-time mental health issues start by age 14, underscoring the substantial importance of childhood and adolescent socioemotional behaviour during the life-course (Kessler *et al*., 2005). The relevance of this issue to public health is shared by policy makers in the UK, as children and young people are targeted as priority groups for mental health promotion (The Mental Health Taskforce to the NHS in England, 2016).

Another important topic is the presence of health inequality. Studies have found worse socioemotional outcomes in children from lower incomes than those from higher incomes (McLeod and Shanahan, 1993; Korenman *et al*., 1995; Green *et al*., 2005; Kelly *et al*., 2011; Sadler *et al*., 2018). Although the number of studies may be less, comparable results have been shown regarding parent's educational or occupational status (von Rueden *et al*., 2006; Perna *et al*., 2010).

A key factor affecting these issues is children's parenting environment. Studies on early to mid-childhood have revealed that negative parenting styles, such as those with harsh, punitive and controlling attitudes, increases the risk of developing socioemotional difficulties, whilst warm and positive parenting has a protective effect for the child (Weiss *et al*., 1992; Gazelle *et al*., 2005; McKee *et al*., 2007; Boeldt *et al*., 2012; van der Sluis *et al*., 2015; Reuben *et al*., 2016). This may also have a long-term impact on children, since some studies indicate that parenting in early childhood is associated with outcomes in pre-adolescence and young adulthood (Beckwith *et al*., 1992; Keiley *et al*., 2001; Lorber and Egeland, 2009; Petersen *et al*., 2015). Considering this longstanding impact and the importance of early child development, a positive early parenting environment could have an impact in reducing the socioeconomic gap between children with regards to their socioemotional outcomes in later stages of life. Studies trying to look at this association suggest that income gradients in children's socioemotional difficulties and competence can be partly explained by parenting activities (Gershoff *et al*., 2007; Kelly *et al*., 2011; Granero *et al*., 2015). Parenting practices were also shown to mediate the association between family economy and children's mental health among adolescents as well (Bøe *et al*., 2014).

Despite these findings, there are several limitations in previous studies. Conger *et al*. point out that most of the previous findings were based on cross-sectional studies, which makes it difficult to discuss long-term effects and causal relationships (Conger *et al*., 2010). Also, they indicate that most of the past studies have constructed a latent factor for socioeconomic position (SEP) or focused solely on income, making it difficult to assess various aspects of SEP. Finally, studies on interaction between SEP and the parenting environment are limited and often small scale. This study aims to overcome some of these limitations and reveal the relationships between SEP, parenting environment and children's behavioural problems in the UK. Based on available evidence, it is hypothesised that: (1) children from disadvantaged SEP are more likely to have worse outcomes throughout childhood and adolescence; (2) parenting environment will attenuate the association between SEP and children's behavioural problems; (3) parenting environment will modify the association between SEP and children's behavioural problems.

## Methods

### Study population and study sample

This study was performed using data from the Millennium Cohort Study (MCS). The MCS is a nationally representative prospective study of children born in the UK between September 2000 and January 2002 (Hansen, 2014). In total, 18 552 families were recruited to the cohort during this period. The first interview was conducted when the children were aged 9 months, and subsequent follow-up interviews were held at ages 3, 5, 7, 11 and 14. Since the main exposures used in this assessment were collected at sweep 1 and 2, those who were not present at these surveys, and those whose main respondents were not their mothers were excluded from this study. Second, since twins and triplets have moderated behavioural characteristics, they were excluded from the sample as well (Hansen and Joshi, 2007). Finally, participants who had never answered the Strengths and Difficulties Questionnaire (SDQ) were excluded. This resulted in a total of 14 452 participants in the whole assessment.

### Socioeconomic position

There are three SEP variables: household income, maternal education and maternal occupation used as the main explanatory variable in the analysis. All the SEP variables were taken from sweep 1 collected through the main respondent's interview when the cohort members were aged 9 months. Household income was categorised into quintiles from the lowest to the highest after being equivalised by the modified Organisation for Economic Co-operation and Development scales (Hansen, 2014). Data on maternal education were measured using the five category National Vocational Qualification (NVQ) classification, alongside with categories defining ‘overseas qualification’ and ‘no qualification’ (Macratos, 2016). NVQ is a competence-based qualification built on UK national occupational standards, where level-1 is the lowest level which involves the application of knowledge and skills for routine and predictable works, and level-5 being the highest level which involves the application of skills and significant range of fundamental principles and complex techniques with substantial autonomy and significant responsibility for the work of others. Occupational class was categorised into six categories, which is ‘managerial and professional’, ‘intermediate’, ‘small employer and self-employed’, ‘lower supervisory and technical’, ‘semi-routine and routine’ and ‘not doing any paid work’, based on the National Statistics Socioeconomic Classification (NS-SEC) (Office for National Statistics, 2005). The NS-SEC is a widely used classification in the UK, and it aims to differentiate positions within labour markets in terms of their employment relations (e.g. *routine occupations*, such as waiters, have the least need for employee discretion and employees are regulated by a basic labour contract).

### Parenting environment

There are 17 items from three domains of parenting domains used in the analysis: ‘learning activities’, ‘family routines’ and ‘psychosocial environment’. These variables were chosen based on previous research and were obtained in sweep 2 through the main respondent's interview (Kelly *et al*., 2011).

#### Learning activities

A total of eight questions were asked regarding the child's learning activities. The questions were collected based on the ‘home learning environment index’ developed by the Effective Provision of Pre-school Education (EPPE) study conducted in England (Melhuish *et al*., 2008; De La Rochebrochard, 2012). Whether someone reads to the child or helped the child learn sport was answered in binary terms; ‘Yes’ or ‘No’. Other questions on learning activities were the frequency of involvement in reading, learning alphabets, counting, singing and painting which were answered in eight ordered categories ranging from ‘do not’ to ‘7 times a week/constantly’.

#### Family routines

Family routines were measured by how often the child went to bed or had meals on a routine time schedule. The two items are also from the EPPE study, and the answers are categorised in five levels ranging from ‘missing’ to ‘always’ (Johnson *et al*., 2015).

#### Psychosocial environment

The child's psychosocial environment was measured by the following seven markers. Mother's parenting competence in five categories ranging from ‘not very good at being a parent’ to ‘a very good parent’. Parent distress measured by Kessler's Psychological distress scale (K6), which is a widely used measure of non-specific psychological distress (Kessler *et al*., 2002; Mitchell and Beals, 2011). Child–parent relationship measured by the scale developed by Pianta (CPRS) (Pianta, 1992). Family rules based on questions on the number of rules and how they were enforced. A composite score based on nine items from the Home Observation for Measurement of the Environment(HOME) inventory (Linver *et al*., 2004), and discipline practices constructed by seven items from the Straus's conflict tactics scale (Straus and Hamby, 1997). The nine items taken from the HOME inventory were whether: the mother's voice was positive when they were speaking to the child; the mother converses at least twice with the child; the mother answers to the child's questions verbally; the mother praises the child spontaneously; the mother caresses or kisses the child; the mother introduces the interviewer to the child; the mother scolded the child more than once; the mother used physical restraint on child; the mother slapped or spanked the child (Chronbach^′^*s α* = 0.6). Similarly, the seven items taken from the Straus's conflict tactics scale were how often the mothers: ignored their child; smacked the child; shouted at the child; sent the child to the bedroom/naughty chair; took away treats; told them off; bribed the child with sweets (Chronbach^′^*s α* = 0.7).

### Demographic indicators

Child's sex and items which may be associated with both household/maternal SEP and children's behavioural problems such as ‘mother's age at the time of birth’, ‘whether the child was a first-born’, and the ‘language spoken at home’ were included as demographic variables, based on previous research, to reduce possibilities of confounding (Kelly *et al*., 2011). Also, the child's age was used as the time-variant variable in the analysis.

### Behavioural problems

Children's behavioural problems were measured using the SDQ score from sweeps 2 to 6. The SDQ is a brief behavioural screening questionnaire widely used to identify children's psychological morbidity and to assess their behavioural problems (Goodman, 1997). It is used for children aged 3–16 years old and is constructed by a total of 25 questions from five domains of behaviour: emotional symptoms, conduct problems, hyperactivity/inattention, peer relationship problems and prosocial behaviours. Since the interest of the study is the behavioural problem of the general population, the scores were sub-scaled into ‘externalising problems’ and ‘internalising problems’ (Goodman *et al*., 2010). Externalising problems generally represent behavioural aspects of children's behavioural problems such as aggression and disruption, while internalising problems stand for emotional aspects such as anxiety and depression. Scores for both externalising and internalising problems range from 0 to 20, and lower score suggests positive outcomes.

### Data management

Missing data in the study sample were multiply imputed using multiple imputation by chained equations (MICE) (Azur *et al*., 2011). All the variables used in the regression analysis were introduced to the imputation model, including the dependent, independent and design variables considering the clustered nature of the data. Imputed values of the dependent variable were excluded afterwards, because the growth curve model can handle unbalanced data and complete data were not required for the dependent variable. Since the amount of missing data ranged from 0 to 20% among the variables, 20 data sets were generated by MICE (White *et al*., 2011). The results of analysis using the imputed data sets were consolidated using Rubin's combination rules (Rubin, 2004). Also, a stratum variable was included in the regression analysis to take into account the stratified sampling process of the MCS. This was based on the suggestion by the Centre of Longitudinal studies, responsible for the MCS (Hansen, 2014).

### Analytical strategy

The children's trajectory in behavioural problems was investigated by fitting a growth curve model with two levels (Table 1). The level-1 sub-model represents how the SDQ scores for child *i* changes by time *j*. It is constructed with parameter *π*_0*i*_ representing the intercept of the individual, and *π*_1*i*_/*π*_2*i*_ representing the linear/quadratic slope of the change trajectory. The level-2 sub-model represents how this trajectory may differ between individuals. The intercept (*π*_0*i*_) and the slope of the change trajectory (*π*_1*i*_/*π*_2*i*_) are considered as level-2 outcomes for each component of the model, and its association with predicting variables is assessed.

**Table 1.** Summary of the models

Model	Level-1 (within individual)	Level-2 (between individual)
1	*y*_*ij*_ = *π*_0*i*_ + ɛ_*ij*_	*π*_*oi*_ = *Intercept*_00_ + *ζ*_0*i*_
2	*y*_*ij*_ = *π*_0*i*_ + *π*_1*i*_*AGE* + *π*_2*i*_*AGE*^2^ + ɛ_*ij*_	*π*_*oi*_ = *Intercept*_0_ + *DEMOGRAPHICS* + *ζ*_0*i*_, *π*_1*i*_ = *Intercept*_1_ + *DEMOGRAPHICS* + *ζ*_1*i*_, *π*_2*i*_ = *Intercept*_2_ + *DEMOGRAPHICS* + *ζ*_2*i*_
3	*y*_*ij*_ = *π*_0*i*_ + *π*_1*i*_*AGE* + *π*_2*i*_*AGE*^2^ + ɛ_*ij*_	*π*_*oi*_ = *Intercept*_0_ + *DEMOGRAPHICS* + *SEP* + *ζ*_0*i*_, *π*_1*i*_ = *Intercept*_1_ + *DEMOGRAPHICS* + *SEP* + *ζ*_1*i*_, *π*_2*i*_ = *Intercept*_2_ + *DEMOGRAPHICS* + *SEP* + *ζ*_2*i*_
4	*y*_*ij*_ = *π*_0*i*_ + *π*_1*i*_*AGE* + *π*_2*i*_*AGE*^2^ + ɛ_*ij*_	*π*_*oi*_ = *Intercept*_0_ + *DEMOGRAPHICS* + *SEP* + *PARENTING* + *ζ*_0*i*_, *π*_1*i*_ = *Intercept*_1_ + *DEMOGRAPHICS* + *SEP* + *PARENTING* + *ζ*_1*i*_, *π*_2*i*_ = *Intercept*_2_ + *DEMOGRAPHICS* + *SEP* + *PARENTING* + *ζ*_2*i*_
5	*y*_*ij*_ = *π*_0*i*_ + *π*_1*i*_*AGE* + *π*_2*i*_*AGE*^2^ + ɛ_*ij*_	*π*_*oi*_ = *Intercept*_0_ + *DEMOGRAPHICS* + *SEP* + *PARENTING* + *SEP* × *PARENTING* + *ζ*_0*i*_, *π*_1*i*_ = *Intercept*_1_ + *DEMOGRAPHICS* + *SEP* + *PARENTING* + *SEP* × *PARENTING* + *ζ*_1*i*_, *π*_2*i*_ = *Intercept*_2_ + *DEMOGRAPHICS* + *SEP* + *PARENTING* + *SEP* × *PARENTING* + *ζ*_2*i*_

The assessment proceeded in five steps shown in Table 1. First, the unconditional means model was fit to assess where the systematic variation resided. Second, the time-related predictors and demographic variables were added to the model, and the general trajectory in children's behavioural problem was investigated. The third step introduced SEP variables to the model, and an assessment was carried on addressing how the trajectory in behavioural problems differed between children with different SEP. The fourth step introduced parenting variables to the model. The variables were chosen based on previous research (Kelly *et al*., 2011), and it was investigated to what extent they may account for the association between SEP and children's behavioural problems. The last step added the interaction term between SEP and parenting variables to identify any interaction between these variables. The interaction terms were introduced to the model in a stepwise way; they were added and excluded from the model one by one and kept in the model if the coefficient was statistically significant at the 0.05 level. The model was fit centring age at 3 when the parenting variables were collected, and at 14 to investigate the effect in adolescence. All the statistical assessment was carried out by Stata 15 (StataCorp, 2017).

## Results

The descriptive statistics of the data are summarised in Appendix 1 and Appendix 2. In general, children with disadvantaged household/maternal SEP had a higher mean SDQ score. The SDQ score for externalising problems generally showed a steady decrease from age 3 to 14, while those for internalising problems showed a mild increase after age 5.

### Socioeconomic gradient in children's behavioural problems

Table 2 shows the results for externalising problems and its association with the three SEP variables: household income maternal education and maternal occupation. The general trajectory of children's SDQ score was evaluated by model-2, showing that the scores tend to decrease in early childhood, but would slightly increase in later childhood and adolescence. Model-3 shows that children from an advantaged household/maternal SEP tend to have lower SDQ scores. For example, the SDQ score of children from the 5th income quintile was −1.25 (95% confidence interval −1.47 to −1.03) lower than those from the lowest quintile. Similarly, children whose mothers were NVQ level 5 had −1.58 (95% confidence interval −1.02 to −1.25) lower scores than those with mothers of no educational qualification. The linear/quadratic rate of change suggests that these differences may narrow during early childhood, but widen thereafter.

**Table 2.** Growth model for externalising problems (centred at age 3)

		Model-1	Model-2	Model-3	Model-4
Fixed effects: coefficients (standard errors)					
Initial status, *π*_0*i*_	Intercept	5.20** (0.026)	10.26** (0.17)	10.32** (0.19)	18.72** (0.46)
	Household income				
	Lowest quintile			–	–
	Second quintile			−0.54** (0.084)	−0.20* (0.068)
	Third quintile			−0.96** (0.095)	−0.43** (0.077)
	Fourth quintile			−1.11** (0.10)	−0.52** (0.083)
	Fifth quintile			−1.25** (0.11)	−0.64** (0.092)
	Maternal education				
	None of below			–	–
	Overseas quality only			−0.31 (0.19)	−0.29 (0.16)
	NVQ level 1			−0.38* (0.12)	−0.19 (0.097)
	NVQ level 2			−0.91** (0.93)	−0.48** (0.077)
	NVQ level 3			−1.14** (0.11)	−0.63** (0.087)
	NVQ level 4			−1.48** (0.10)	−0.95** (0.086)
	NVQ level 5			−1.58** (0.17)	−1.12** (0.14)
	Maternal occupation				
	Not in work/on leave			–	–
	Semi-routine and routine			0.29* (0.86)	0.21* (0.069)
	Lower supervisory and technical			0.11 (0.17)	0.24 (0.14)
	Small employers and self-employed			−0.58* (0.17)	−0.32* (0.14)
	Intermediate			−0.060 (0.96)	0.058 (0.076)
	Managerial and professional			−0.21* (0.087)	−0.0097 (0.068)
Linear rate of change, *π*_1*i*_	Intercept		−0.79** (0.055)	−0.80** (0.062)	−2.10** (0.19)
	Household income				
	Lowest quintile			–	–
	Second quintile			0.0085 (0.028)	−0.018 (0.028)
	Third quintile			0.0097 (0.031)	−0.032 (0.031)
	Fourth quintile			−0.0092 (0.034)	−0.052 (0.034)
	Fifth quintile			0.0088 (0.037)	−0.037 (0.037)
	Maternal education				
	None of below			–	–
	Overseas quality only			0.049 (0.064)	0.069 (0.062)
	NVQ level 1			0.066 (0.040)	0.056 (0.039)
	NVQ level 2			0.081* (0.032)	0.059 (0.031)
	NVQ level 3			0.078* (0.036)	0.057 (0.036)
	NVQ level 4			0.11* (0.035)	0.096* (0.035)
	NVQ level 5			0.15* (0.056)	0.14* (0.055)
	Maternal occupation				
	Not in work/on leave			–	–
	Semi-routine and routine			−0.012 (0.028)	−0.010 (0.028)
	Lower supervisory and technical			0.064 (0.056)	0.056 (0.055)
	Small employers and self-employed			0.12* (0.056)	0.10 (0.055)
	Intermediate			0.0081 (0.031)	−0.0054 (0.030)
	Managerial and professional			0.049 (0.028)	0.023 (0.027)
Quadratic rate of change, *π*_2*i*_	Intercept		0.058** (0.0051)	0.058** (0.0057)	0.13** (0.018)
	Household income				
	Lowest quintile			–	–
	Second quintile			−0.0022 (0.0026)	−0.00074 (0.0026)
	Third quintile			−0.0027 (0.0029)	−0.00040 (0.0029)
	Fourth quintile			−0.00079 (0.0031)	0.0014 (0.0031)
	Fifth quintile			−0.0014 (0.0034)	0.0011 (0.0034)
	Maternal education				
	None of below			–	–
	Overseas quality only			−0.0033 (0.0058)	−0.0048 (0.0057)
	NVQ level 1			−0.0028 (0.0037)	−0.0026 (0.0037)
	NVQ level 2			−0.0045 (0.0029)	−0.0037 (0.0030)
	NVQ level 3			−0.0045 (0.0033)	−0.0039 (0.0034)
	NVQ level 4			−0.0076* (0.0032)	−0.0076* (0.0033)
	NVQ level 5			−0.011* (0.0051)	−0.012* (0.0051)
	Maternal occupation				
	Not in work/on leave			–	–
	Semi-routine and routine			0.00058 (0.0026)	0.00056 (0.0026)
	Lower supervisory and technical			−0.0062 (0.0052)	−0.0059 (0.0051)
	Small employers and self-employed			−0.0065 (0.0052)	−0.0053 (0.0051)
	Intermediate			0.00028 (0.0029)	0.0012 (0.0028)
	Managerial and professional			−0.0038 (0.0026)	−0.0022 (0.0025)
Variance components: standard deviations and correlation coefficients (standard errors)					
Level-1	Within-person (*σ*_ɛ_)	2.49 (0.0084)	1.97 (0.0096)	1.97 (0.0096)	1.97 (0.0096)
Level-2	Initial status (*σ*_0_)	2.76 (0.020)	2.86 (0.022)	2.76 (0.022)	1.94 (0.019)
	Linear rate of change (*σ*_1_)		0.60 (0.012)	0.59 (0.012)	0.54 (0.012)
	Corr (linear – initial)		−0.26 (0.015)	−0.25 (0.015)	0.057 (0.022)
	Quadratic rate of change (*σ*_2_)		0.045 (0.0013)	0.045 (0.0013)	0.042 (0.0013)
	Corr (quadratic – initial)		0.12 (0.019)	0.11 (0.020)	−0.20 (0.027)
	Corr (quadratic – linear)		−0.94 (0.0035)	−0.94 (0.0035)	−0.93 (0.0042)
Pseudo *R*^2^ statistics					
	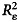		0.38	0.38	0.37
	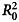			0.07	0.54
	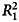			0.007	0.19
	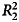			0.006	0.13

*p < 0.05, **p < 0.001.

Model-1: empty.

Model-2: demographics.

Model-3: demographics + SEP.

Model-4: demographics + SEP + parenting.

Table 3 shows the results for internalising problems and its association with the three SEP variables: household income, maternal education and maternal occupation. Model-2 shows that the decreasing trend in the early ages was much milder and shorter in internalising problems, indicating a generally increasing trend in SDQ scores. As in externalising problems, model-3 shows that children with advantaged household/maternal SEP tend to have lower SDQ scores. For example, children from the 5th income quintile had lower scores by −0.82 (95% confidence interval −0.97 to −0.67) compared to those from the lowest quintile. Similarly, children whose mothers were NVQ level 5 had −0.92 (95% confidence interval −1.15 to −0.70) lower scores than those with mothers of no educational qualification. However, as in externalising problems, the difference between maternal occupation was milder than the other two SEP variables.

**Table 3.** Growth model for internalising problems (centred at age 3)

		Model-1	Model-2	Model-3	Model-4	Model-5
Fixed effects: coefficients (standard errors)						
Initial status, *π*_0*i*_	Intercept	3.00** (0.019)	4.13** (0.11)	4.36** (0.12)	10.09** (0.36)	11.39** (0.44)
	Household income					
	Lowest quintile			–	–	–
	Second quintile			−0.32** (0.057)	−0.13* (0.053)	−0.76 (0.43)
	Third quintile			−0.60** (0.064)	−0.26** (0.060)	−2.24** (0.46)
	Fourth quintile			−0.80** (0.069)	−0.41** (0.065)	−2.95** (0.49)
	Fifth quintile			−0.82** (0.076)	−0.42** (0.071)	−2.93** (0.50)
	Maternal education					
	None of below			–	–	–
	Overseas quality only			−0.47** (0.13)	−0.43** (0.12)	−0.43** (0.12)
	NVQ level 1			−0.49** (0.080)	−0.35** (0.074)	−0.34** (0.074)
	NVQ level 2			−0.77** (0.063)	−0.50** (0.059)	−0.48** (0.059)
	NVQ level 3			−0.92** (0.072)	−0.59** (0.068)	−0.58** (0.068)
	NVQ level 4			−1.03** (0.070)	−0.68** (0.067)	−0.68** (0.067)
	NVQ level 5			−0.92** (0.12)	−0.65** (0.11)	−0.64** (0.11)
	Maternal occupation					
	Not in work/on leave			–	–	–
	Semi-routine and routine			−0.013 (0.058)	−0.0066 (0.053)	0.0030 (0.053)
	Lower supervisory and technical			−0.084 (0.11)	−0.0085 (0.11)	−0.0055 (0.11)
	Small employers and self-employed			−0.046 (0.12)	0.11 (0.11)	0.10 (0.11)
	Intermediate			−0.27** (0.064)	−0.18* (0.059)	−0.19* (0.059)
	Managerial and professional			−0.22** (0.058)	−0.10 (0.53)	−0.11* (0.053)
	(Household income) × (CPRS)					
	Lowest quintile					–
	Second quintile					0.010 (0.0067)
	Third quintile					0.031** (0.0071)
	Fourth quintile					0.040** (0.0075)
	Fifth quintile					0.39** (0.0077)
Linear rate of change, *π*_1*i*_	Intercept		−0.18** (0.046)	−0.21** (0.052)	−0.66** (0.16)	−0.83** (0.16)
	Household income					
	Lowest quintile			–	–	–
	Second quintile			−0.0027 (0.024)	0.0055 (0.024)	0.032 (0.081)
	Third quintile			−0.022 (0.026)	−0.013 (0.027)	0.25* (0.087)
	Fourth quintile			−0.041 (0.028)	−0.028 (0.029)	0.35** (0.093)
	Fifth quintile			−0.043 (0.031)	−0.029 (0.032)	0.30* (0.096)
	Maternal education					
	None of below			–	–	–
	Overseas quality only			0.11 (0.53)	0.12* (0.053)	0.12* (0.053)
	NVQ level 1			0.011 (0.34)	0.013 (0.034)	0.013 (0.034)
	NVQ level 2			0.036 (0.027)	0.041 (0.027)	0.039 (0.027)
	NVQ level 3			0.073* (0.030)	0.080* (0.031)	0.079* (0.031)
	NVQ level 4			0.084* (0.029)	0.094* (0.030)	0.093* (0.030)
	NVQ level 5			0.087 (0.047)	0.096* (0.047)	0.094* (0.047)
	Maternal occupation					
	Not in work/on leave			–	–	–
	Semi-routine and routine			0.00093 (0.024)	0.0056 (0.024)	0.0040 (0.024)
	Lower supervisory and technical			0.0075 (0.047)	0.014 (0.047)	0.014 (0.047)
	Small employers and self-employed			0.0070 (0.047)	0.013 (0.047)	0.013 (0.047)
	Intermediate			−0.0049 (0.026)	0.00031 (0.026)	0.00082 (0.026)
	Managerial and professional			−0.021 (0.023)	−0.019 (0.023)	−0.017 (0.023)
	(Household income) × (CPRS)					
	Lowest quintile					–
	Second quintile					−0.00046 (0.0012)
	Third quintile					−0.0042* (0.0013)
	Fourth quintile					−0.0060** (0.0014)
	Fifth quintile					−0.0051* (0.0014)
Quadratic rate of change, *π*_0*i*_	Intercept		0.025** (0.0045)	0.024** (0.0051)	0.049* (0.015)	0.049* (0.015)
	Household income					
	Lowest quintile			–	–	–
	Second quintile			−0.00094 (0.0023)	−0.0016 (0.0024)	−0.0016 (0.0024)
	Third quintile			0.00023 (0.0026)	−0.00053 (0.0026)	−0.00057 (0.0026)
	Fourth quintile			0.00062 (0.0028)	−0.00045 (0.0028)	−0.00049 (0.0028)
	Fifth quintile			−0.00023 (0.0030)	−0.0013 (0.0031)	−0.0014 (0.0031)
	Maternal education					
	None of below			–	–	–
	Overseas quality only			−0.0063 (0.0051)	−0.0071 (0.0051)	−0.0070 (0.0051)
	NVQ level 1			0.0016 (0.0033)	0.0011 (0.0033)	0.0011 (0.0033)
	NVQ level 2			−0.00049 (0.0026)	−0.0015 (0.0027)	−0.0015 (0.0027)
	NVQ level 3			−0.0042 (0.0030)	−0.0055 (0.0030)	−0.0055 (0.0030)
	NVQ level 4			−0.0063* (0.0029)	−0.0079* (0.0030)	−0.0079* (0.0030)
	NVQ level 5			−0.0085 (0.0045)	−0.010* (0.0046)	−0.010* (0.0046)
	Maternal occupation					
	Not in work/on leave			–	–	–
	Semi-routine and routine			−0.00070 (0.0023)	−0.0011 (0.0023)	−0.0011 (0.0023)
	Lower supervisory and technical			−0.00052 (0.0046)	−0.0011 (0.0046)	−0.0012 (0.0046)
	Small employers and self-employed			0.0012 (0.0046)	0.00086 (0.0046)	0.00085 (0.0046)
	Intermediate			0.000062 (0.0025)	−0.00035 (0.0025)	−0.00039 (0.0025)
	Managerial and professional			0.0018 (0.0023)	0.0016 (0.0023)	0.0016 (0.0023)
Variance components: standard deviations and correlation coefficients (standard errors)						
Level-1	Within-person (*σ*_ɛ_)	2.15 (0.0072)	1.77 (0.0086)	1.77 (0.0086)	1.77 (0.0086)	1.76 (0.0086)
Level-2	Initial status (*σ*_0_)	1.91 (0.015)	1.71 (0.017)	1.63 (0.017)	1.39 (0.017)	1.42 (0.017)
	Linear rate of change (*σ*_1_)		0.42 (0.012)	0.42 (0.012)	0.41 (0.012)	0.42 (0.012)
	Corr (linear – initial)		0.075 (0.025)	0.085 (0.025)	0.11 (0.028)	0.12 (0.028)
	Quadratic rate of change (*σ*_2_)		0.037 (0.0012)	0.037 (0.0012)	0.036 (0.0012)	0.036 (0.0012)
	Corr (quadratic – initial)		−0.14 (0.027)	−0.16 (0.028)	−0.19 (0.031)	−0.20 (0.031)
	Corr (quadratic – linear)		−0.83 (0.010)	−0.83 (0.010)	−0.83 (0.010)	−0.83 (0.010)
Pseudo *R*^2^ statistics						
	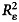		0.33	0.33	0.33	0.33
	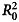			0.08	0.34	0.34
	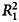			0.007	0.03	0.03
	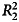			0.005	0.02	0.02

*p < 0.05, **p < 0.001.

Model-1: empty.

Model-2: demographics.

Model-3: demographics + SEP.

Model-4: demographics + SEP + parenting.

Model-5: demographics + SEP + parenting + interaction term.

### Early parenting environment and socioeconomic gradient in children's behavioural problems

**Fig. 1.**
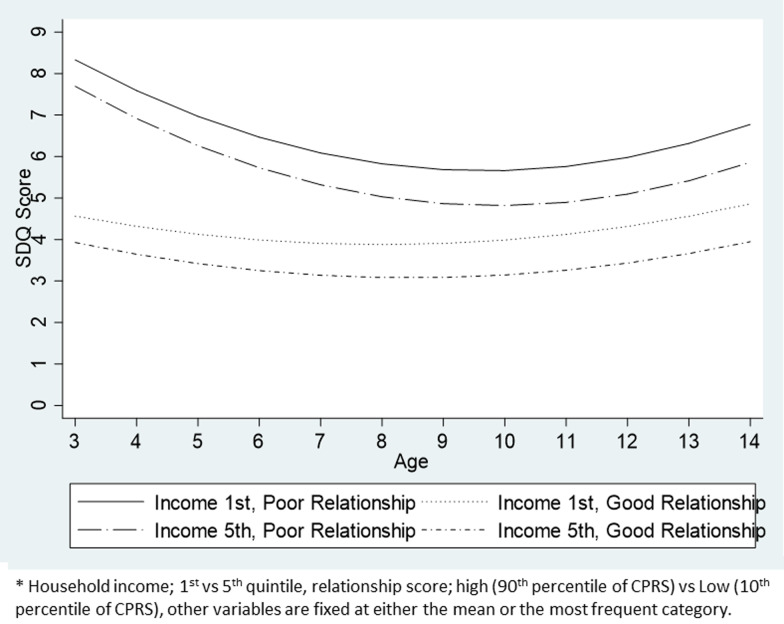
Predicted trajectories of externalising problems by household income and child–parent relationship.

For externalising problems, the introduction of variables for parenting environment attenuated the association between SDQ scores and SEP by 49% for household income, 29% for maternal education and 95% for maternal occupation at age 3, and 31, 26 and 82%, respectively, at age 14 (Table 4). Considering the variance components, there was an additional 47% reduction in the level-2 variance of the initial status (Table 2: model-4). Parenting environment seems to have explained a larger part of the difference in SDQ scores between children than SEP (Fig. 1).

**Table 4.** Attenuation of socioeconomic gradient in children's SDQ score by the introduction of parenting variables

	Externalising problems		Internalising problems	
	Age 3	Age 14	Age 3	Age 14
Household income	−49%	−31%	−49%	−32%
Maternal education	−29%	−26%	−30%	−17%
Maternal occupation	−95%	−82%	−55%	−50%

For internalising problems, the introduction of parenting variables attenuated the association between SDQ scores and SEP by 49% for household income, 30% for maternal education and 55% for maternal occupation at age 3, and 32, 17 and 50%, respectively, at age 14 (Table 4). There was an additional 26% reduction in the level-2 variance of the initial status, showing that parenting environment explained a certain degree of difference in SDQ scores between children (Table 3: model-4).

### Evaluation of interactions

**Fig. 2.**
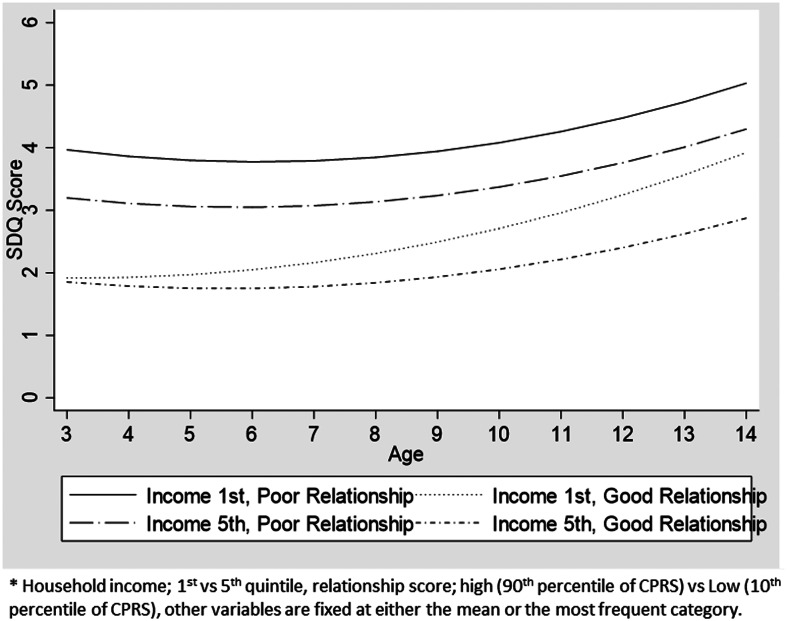
Predicted trajectories of internalising problems by household income and child–parent relationship.

There was no statistically significant interaction between household/maternal SEP and parenting environment with regards to externalising problems. On the contrary, there was a statistically significant interaction regarding internalising problems where parent–child relationship modified the effect of household income (Table 3: model-5). There was a more modest income gradient among those with better parent–child relationship in early childhood. However, the income gradient within this group grew as children aged. By the time they reached adolescence, the difference in SDQ scores between children from 1st and 5th income quintiles was larger within children who were under a better parent–child relationship than those under poor parent–child relationships. Nonetheless, the SDQ score was better for those who had good parent–child relationship than those who had a poor one, irrespective of their SEP (Fig. 2).

## Discussion

### Summary of findings and comparison with other studies

The aim of this study was to reveal the relationships between SEP, parenting environment and children's behavioural problems. For both internalising and externalising problems, children under disadvantaged SEP during early childhood were more likely to have worse behavioural problems. However, the socioeconomic gradient differed between measures of SEP, showing a greater gap between those in advantaged and disadvantaged categories for household income and maternal education, and a smaller gap for maternal occupation. Second, early parenting environment had a stronger independent association with children's socioemotional well-being than parental SEP and also attenuated the socioeconomic gradient throughout childhood and adolescence. Finally, there was an interaction between household income and parent–child relationship for internalising problems. It indicated that good parent–child relationship buffered the income gradient in children's behavioural problems during early childhood, and although this buffering effect did not last until adolescence, those who had good parent–child relationships developed better outcomes regardless of their SEP.

Although previous studies have shown that parenting may attenuate the income gradient in children's behavioural problems, most of these studies have been cross-sectional studies and therefore its temporality and long-term effects were not clear (Gershoff *et al*., 2007; Kelly *et al*., 2011; Bøe *et al*., 2014). Perhaps reflecting the rising influence from other factors such as school activity and peer relationships, this study showed that the effect of attenuation weakened as children aged. Nonetheless, early parenting environment still explained a certain degree of socioeconomic gradient in SDQ scores among adolescents. Another strength of this study is the multiple measures used to assess SEP. A cross-sectional pathway analysis conducted on Norwegian adolescents showed that maternal education did not have a direct effect on either internalising or externalising problems after introducing parenting variables, while household income had a direct association with internalising problems (Bøe *et al*., 2014). This is somewhat different from this study, where both SEP variables preserved a significant association with behavioural problems. Since the model in the Norwegian study only explained half the variance of the children's behavioural problems explained in this study, this difference may be due to a smaller amount of inequality and a powerful social security system in the Nordic countries. Finally, this study assessed whether there was an interaction between SEP and parenting environment, finding evidence in internalising problems. Harsh parenting, for which some studies found significant interaction, was not shown to moderate the effect, possibly because of the broader aspects of parenting covered in this study (MacKenzie *et al*., 2014; Flouri and Midouhas, 2017). For example, Flouri and Midouhas investigated the roll of harsh parenting in moderating the effects of socioeconomic disadvantage and adverse life events on children's behavioural problems, and have found some evidence of support (Flouri and Midouhas, 2017). However, since their interest was focused on harsh parenting, they did not include much variables to control for other parenting activities which contrasts to this study. The interaction on internalising problems found in this study was consistent with a former study which showed that parent–child relationship buffered the socioeconomic disadvantage in 3-year-old children (Malmberg and Flouri, 2011). However, this study has revealed that this buffering effect declines as children grow. Since parenting data were taken when children were 3 years old, this decrease in the buffering effect might possibly be reflecting the difficulty of maintaining good parent–child relationship in disadvantaged families.

### Meaning of the study

In general, this study has shown that a low-quality parenting environment in early childhood is a considerable risk for developing behavioural problems, regardless of children's familial/maternal SEP. Therefore, it is crucial to improve the parenting environment to prevent children from developing behavioural problems. Interventions such as home visiting programmes and community programmes providing parents with support and guidance may contribute to improving this environment, and at the end, this might also narrow the socioeconomic gradient among them. Second, for those programmes and interventions which take targeted strategies, this study suggests that in childhood, children in lower parenting environment are under higher risk than those from disadvantaged SEP families. However, as children age, those from lower household income may require consideration, because they will be under increased risk in developing internalising problems even if they were under favourable parenting environment. Finally, when considering importance between policies to improve children's socioeconomic circumstances, this study suggests that policies focusing on maternal occupation may be less important in raising their behavioural outcomes. Furthermore, since household income seems to have more effect than maternal education as children grow older, income-related policies may require the most importance in the long run.

### Strengths and limitations

A distinct strength of this study is that it is a prospective longitudinal analysis, based on large-scale samples from a nationally representative cohort. This makes the temporality of the association clearer and the external validity stronger. The number of sweeps and variables collected in the MCS has also led to strengths. It has contributed in reducing the possibility of residual bias and enabled this study to apply a growth curve model which, compared to traditional techniques, has an advantage in handling missing data and modelling trajectories in the individual level rather than the aggregate level (StataCorp, 2017). Also, data for MCS were collected through trained interviewers to improve the quality of the data. On the contrary, there are some limitations. One of them is regarding the selection of the study sample and missing data. The initial response rate of the MCS ranged between 60 and 70%, which may cause bias in the result of the study (Hansen, 2014). However, evaluation of the known characteristics in those lost before issue to field did not seem to be systematically biased (Plewis *et al*., 2007; Hansen, 2014). Also, missing data within the study sample were either accounted for by the growth curve model or by multiple imputation. Another possible limitation of this study was that the SDQ scores used in this study were based on parent report. In a previous study using the MCS, the socioeconomic gradient in the SDQ score was stronger in parent report compared to teacher report, which may be demonstrating reporting bias (Plewis *et al*., 2007). Nevertheless, the socioeconomic gradient was statistically significant in both parent and teacher report, and the scores were well correlated in their study. Also, SDQ is widely known as a validated and reliable measure of children's behavioural status (Goodman, 2001; Plewis *et al*., 2007).

Some topics which were not covered by this study may provide implications for further research. Regarding SEP, this study did not cover paternal measures. Some study indicates that paternal SEP may have an independent effect on children's behavioural problems, and may have a pathway different from those for maternal SEP (Bøe *et al*., 2014). Also, further follow-up may be important to assess the long-term effect of parenting environment, since some small-scaled study found a rebound in the strength of association after adolescence (Lorber and Egeland, 2009). Finally, although early parenting explained a certain degree of gradient in behavioural problems of adolescents, much of the variance is still unexplained. Further research may be called for to reveal how this socioeconomic gradient is fully explained.

## Data Availability

The full dataset used for this study is available from the UK Data Service under standard conditions: https://discover.ukdataservice.ac.uk/series/?sn=2000031#access.

## Appendix

**Appendix 1.** Descriptive data for exposures

	*N* (%)	Mean (s.d.)
Mother's age at child's birth		
Missing	2 (0.01)	–
Valid	14 450 (99.99)	28.66 (5.87)
Child's sex		
Male	7388 (51.12)	
Female	7064 (48.88)	
First child		
Not first child	8458 (58.52)	
First child	5994 (41.48)	
Household language		
Missing	67 (0.46)	
English only	12 267 (84.88)	
Any other language spoken	2118 (14.66)	
Household income		
Missing	38 (0.26)	
Lowest quintile	3235 (22.38)	
Second quintile	3113 (21.54)	
Third quintile	2799 (19.37)	
Fourth quintile	2728 (18.88)	
Fifth quintile	2539 (17.57)	
Maternal education		
Missing	21 (0.15)	
None of below	2055 (14.22)	
Overseas quality only	384 (2.66)	
NVQ level 1	1193 (8.25)	
NVQ level 2	4174 (28.88)	
NVQ level 3	2097 (14.51)	
NVQ level 4	4015 (27.78)	
NVQ level 5	513 (3.55)	
Maternal occupation		
Missing	71 (0.49)	
Not in work/on leave	7288 (50.43)	
Semi-routine and routine	1860 (12.87)	
Lower supervisory and technical	362 (2.50)	
Small employers and self-employed	351 (2.43)	
Intermediate	1539 (10.65)	
Managerial and professional	2981 (20.63)	
Mother's reading		
Missing	68 (0.47)	
Every day	8279 (57.29)	
Several times a week	2769 (19.16)	
Once or twice a week	2233 (15.45)	
Once or twice a month	374 (2.59)	
Less often	288 (1.99)	
Not at all	441 (3.05)	
Anyone else reading		
Missing	68 (0.47)	
Yes	12 196 (84.39)	
No	2188 (15.14)	
Helping with sports		
Missing	68 (0.47)	
Yes	11 349 (78.53)	
No	3035 (21.00)	
Taking to the library		
Missing	68 (0.47)	
Do not	8554 (59.19)	
On special occasions	1327 (9.18)	
Once a month	2416 (16.72)	
Once a fortnight	1084 (7.50)	
Once a week	1003 (6.94)	
Helping with alphabet		
Missing	69 (0.48)	
Do not	2766 (19.14)	
Occasionally/less than once a week	1810 (12.52)	
1–2 days per week	3128 (21.64)	
3 times a week	1795 (12.42)	
4 times a week	1073 (7.42)	
5 times a week	706 (4.89)	
6 times a week	343 (2.37)	
7 times a week/constantly	2762 (19.11)	
Helping with counting		
Missing	69 (0.48)	
Do not	547 (3.78)	
Occasionally/less than once a week	752 (5.20)	
1–2 days per week	1949 (13.49)	
3 times a week	1540 (10.66)	
4 times a week	1129 (7.81)	
5 times a week	936 (6.48)	
6 times a week	605 (4.19)	
7 times a week/constantly	6925 (47.92)	
Teaching songs		
Missing	69 (0.48)	
Do not	665 (4.60)	
Occasionally/less than once a week	560 (3.87)	
1–2 days per week	1512 (10.46)	
3 times a week	1316 (9.11)	
4 times a week	1097 (7.59)	
5 times a week	1008 (6.97)	
6 times a week	617 (4.27)	
7 times a week/constantly	7608 (52.64)	
Child drawing		
Missing	69 (0.48)	
Do not	286 (1.98)	
Occasionally/less than once a week	566 (3.92)	
1–2 days per week	2042 (14.13)	
3 times a week	1716 (11.87)	
4 times a week	1434 (9.92)	
5 times a week	1227 (8.49)	
6 times a week	801 (5.54)	
7 times a week/constantly	6311 (43.67)	
Regular bed times		
Missing	68 (0.47)	
Never/almost never	1112 (7.69)	
Sometimes	1988 (13.76)	
Usually	5417 (37.48)	
Always	5867 (40.60)	
Regular meal times		
Missing	68 (0.47)	
Never/almost never	306 (2.12)	
Sometimes	1083 (7.49)	
Usually	6166 (42.67)	
Always	6829 (47.25)	
Family rules		
Missing	69 (0.48)	
Lots of rules	4201 (29.07)	
Not many rules	6292 (43.54)	
It varies	3890 (26.92)	
Enforcement of rules		
Missing	69 (0.48)	
Strictly enforced	6601 (45.68)	
Not very strictly enforced	3806 (26.34)	
It varies	3976 (27.51)	
Parenting competence		
Missing	1619 (11.20)	
Not very good	36 (0.25)	
Have some trouble being a parent	371 (2.57)	
Average parent	4844 (33.52)	
Better than average parent	3290 (22.77)	
Very good parent	4292 (29.70)	
K6 score		
Missing	1511 (10.46)	–
Valid	12 941 (89.54)	3.25 (3.74)
CPRS		
Missing	1649 (11.41)	–
Valid	12 803 (88.59)	64.20 (7.05)
HOME inventory		
Missing	1892 (13.09)	–
Valid	12 560 (86.91)	9.65 (1.07)
Discipline strategy		
Missing	2868 (19.85)	–
Valid	11 584 (80.15)	19.98 (5.00)

**Appendix 2.** Descriptive data for outcomes (mean SDQ scores) by SEP

	Age 3	Age 5	Age 7	Age 11	Age 14
*Internalising problems*					
Household income					
Lowest quintile	3.88	3.37	3.67	4.13	4.84
Second quintile	3.28	2.82	3.10	3.64	4.24
Third quintile	2.72	2.31	2.57	3.07	3.59
Fourth quintile	2.31	1.97	2.11	2.59	3.09
Highest quintile	2.20	1.91	2.05	2.47	2.84
Maternal education					
None of below	4.17	3.58	3.85	4.16	4.81
Overseas quality only	3.64	3.29	3.63	3.95	4.68
NVQ level 1	3.46	3.04	3.28	3.74	4.59
NVQ level 2	2.96	2.47	2.72	3.31	3.89
NVQ level 3	2.70	2.30	2.54	3.10	3.59
NVQ level 4	2.29	1.99	2.16	2.60	3.02
NVQ level 5	2.32	2.11	2.09	2.48	2.77
Maternal occupation					
Not in work/on leave	3.33	2.84	3.13	3.58	4.21
Semi-routine and routine	3.04	2.63	2.83	3.38	3.83
Lower supervisory and technical	2.75	2.27	2.68	3.14	3.73
Small employers and self-employed	2.58	2.26	2.44	2.96	3.63
Intermediate	2.42	2.12	2.25	2.78	3.29
Managerial and professional	2.19	1.89	2.00	2.48	2.90
*Externalising problems*					
Household income					
Lowest quintile	8.25	6.09	5.99	5.78	5.65
Second quintile	7.30	5.26	5.21	4.96	4.86
Third quintile	6.53	4.50	4.54	4.32	4.05
Fourth quintile	5.88	4.14	4.03	3.71	3.66
Highest quintile	5.39	3.71	3.71	3.39	3.31
Maternal education					
None of below	8.39	6.22	5.92	5.64	5.52
Overseas quality only	7.67	5.54	5.56	4.89	5.02
NVQ level 1	7.96	5.81	5.84	5.55	5.61
NVQ level 2	6.98	4.99	4.90	4.78	4.60
NVQ level 3	6.57	4.60	4.58	4.29	4.19
NVQ level 4	5.61	3.83	3.85	3.55	3.38
NVQ level 5	5.07	3.58	3.61	3.17	3.13
Maternal occupation					
Not in work/on leave	7.31	5.25	5.16	4.89	4.80
Semi-routine and routine	7.27	5.12	5.11	4.90	4.67
Lower supervisory and technical	6.65	4.88	4.88	4.41	4.40
Small employers and self-employed	5.44	3.89	4.11	3.75	3.90
Intermediate	6.33	4.33	4.38	4.19	4.03
Managerial and professional	5.42	3.79	3.73	3.47	3.29
